# Universal behaviour of high-*Q* Fano resonances in metamaterials: terahertz to near-infrared regime

**DOI:** 10.1186/s40580-018-0137-2

**Published:** 2018-02-21

**Authors:** Wen Xiang Lim, Ranjan Singh

**Affiliations:** 1 Division of Physics and Applied Physics, School of Physical and Mathematical Sciences, Nanyang Technological University, Singapore, 637371 Singapore; 2 Centre for Disruptive Photonic Technologies, The Photonics Institute, Nanyang Technological University, 50 Nanyang Avenue, Singapore, 639798 Singapore

**Keywords:** Fano resonance, Metamaterials, Quality factor, Exponential decay, Universal system, Scalability

## Abstract

The observation of Fano resonance phenomena is universal across several branches of physics. Photonics is one of the most important areas of physics that mainly deals with the control of light propagation and localization through its interaction with natural and artificially engineered media. In an era of miniaturization, manipulation of light at micro-nanoscales has assumed unprecedented significance due to its potential to satisfy the mankind with disruptive future technologies. In this work, we present our study on the universality of high quality factor Fano resonances in planar metamaterials across terahertz and infrared parts of the electromagnetic spectrum. The narrow linewidth asymmetric Fano resonant metamaterials have tremendous potential to find applications in micro-nanoscale flat lasers, sensors, and ultra-resolution spectrometers.

## Introduction

In recent years, the field of plasmonics and metamaterials have enabled a seemingly new and innovative direction in the optics and photonics community, promising the conceptualization of subwavelength inventions with advanced and exceptional functionalities [1–3]. Metamaterials [4] are composed of periodic arrangements of artificially engineered meta-atoms whose optical and physical properties are determined by its size, shape and geometry. Meta-atoms are the building blocks of the metamaterials with artificial properties of atoms. When the ensembles of meta-atoms interact with the incident electromagnetic waves, fascinating optical and physical properties non-existent in natural materials are manifested, which allow them to have the ability to guide, modulate or slow down light. The occurrence of strong light-matter interactions in metallic nano- to micro-structures is beneficial for applications in sensors [5–8], absorbers [9, 10], nanolasers [11], photoswitches [11–15, 16] and slow-light devices [17]. However, metallic subwavelength structures entail extremely high losses due to absorption, especially at higher frequency regime of the electromagnetic spectrum where the energies are transferred into optical and acoustic phonons [18]. Radiative and non-radiative losses render the operation of metamaterial devices inefficient and hence impractical. As such, several solutions were proposed to reduce the inherent losses dominant in metamaterials which involve replacing metals with highly doped semiconductors [19] or superconductors [20], or to compensate losses by integrating metamaterials with optical gain medium [21, 22].

Apart from introducing new plasmonic materials or optical gain into the system, geometric tailoring of the subwavelength structures offers an additional platform to mitigate radiative losses. In general, the symmetry of a metamaterial system exhibits a broad dipole resonance that can be excited by the far-field of the propagating electromagnetic wave in free space. However, breaking the symmetry of a unit cell induces a sharp asymmetric lineshape which is forbidden in a perfectly symmetric system. This sharp asymmetric lineshape which is also known as Fano resonance [23] arises from the destructive interference between a bright (continuum) mode and a dark (discrete) mode. The bright mode is superradiant and appears as a broad dipole resonance in the spectrum. As the bright mode has a finite dipole moment whose polarization of the conduction electrons are aligned similarly to the polarization of the electromagnetic field, it can strongly couple with the electromagnetic field. Therefore, energies are transferred between the conduction electrons and the oscillating fields of the propagating electromagnetic wave which are then scattered to the far-field, contributing to the radiative losses in the system. The dark mode which is subradiant in nature, can only be excited indirectly through near-field coupling with the superradiant bright mode, which in turn interferes back with the superradiant bright mode to produce Fano resonance. This process suppresses the radiative losses in Fano resonators and the overall losses are mainly contributed by non-radiative losses due to resistive heating (especially at high frequencies). In metals, free conduction electrons are constantly moving in random motion among the cloud of positive lattice ions. When the free conduction electrons collide with the lattice ions or another free electron, energy is lost non-radiatively to the environment in the form of heat due to resistive heating in metallic Fano resonators. However, at low frequencies, Drude metals have a higher conductivity and so non-radiative losses can be reduced, which leads to radiative losses contributing as the main loss mechanism. In summary, the sharp asymmetric lineshape of Fano resonance is more prominent at terahertz frequency as compared to infrared frequency due to lower non-radiative losses.

Numerous designs of the unit cell in Fano system have been proposed, whereby the symmetry of the unit cell is broken either (in a single-particle system) by altering the geometry of the unit cell [24] or (in a dual-particle system) by moving one of the sub-unit cell [25, 26] or modifying the dimension/geometry of the sub-unit cell [27–29] relative to the other neighbouring sub-unit cell to induce the sharp asymmetric lineshape. Fano resonance has also been demonstrated in multi-particle system such as the dolmen structure (the unit cell itself possesses asymmetry) [30], or in the heptamer (a special case of symmetric structure) [31, 32] whereby Fano resonance is a result of out-of-phase interactions between the bodies of particles. The degree of asymmetry of the unit cell is determined by asymmetry parameter which is a dimensionless parameter and it is defined relative to its specific Fano system. In addition, Fano resonance is most easily excited in positive Fano structures when the polarization of the electric field incident onto the unit cell is parallel to the symmetry breaking axis of the unit cell.

Fano resonance in optical metamaterial system is highly attractive, primarily because at the resonance frequency, it has strong electromagnetic field confinement within the structures and an extremely narrow linewidth which also dictates its quality (*Q*) factor. *Q*-factor is often used as a dimensionless parameter to characterize the strength of damping on the metamaterial resonators. For a metamaterial system with heavy damping, the resonance will not be able to sufficiently survive the energy lost through radiative and non-radiative pathways which cause the resonance linewidth to be largely broadened, and vice versa. Therefore, *Q*-factor helps to determine how much of the energy is being lost or confined, which allows further refinements to be made to optimize the performance of the metamaterial system. Albeit the abundance of discussion on Fano system, there remains a void in the systematization of the *Q*-factors for different asymmetry parameters in complementary structures across the near-infrared (NIR) to terahertz (THz) regime. It has been observed in several works that the *Q*-factor of Fano system decays exponentially with increasing asymmetry parameter [33–35]. However, the similarity in the decay trend of the *Q*-factor ubiquitous in Fano system has received less attention and remains an essential part of understanding the implications of radiative and non-radiative losses. Hence, if a standard system of decay trends under different situations could be developed, it could serve as a guide for appropriate optimization and fabrication of scalable and functional metamaterial-based devices. In this work, we performed numerical calculations on a complementary asymmetric dipole bars, and by proportionally scaling the dimensions we induced Fano resonance at different frequency regimes ranging from NIR to THz. The *Q*-factors at different regimes were evaluated and fitted with an exponential decay function to extract the decay constants. Our results further show that for the smallest asymmetry parameter, the decay constant of the *Q*-factor obtained as a function of the Fano resonance frequency from NIR to THz regime is the largest. We also observed that the decay constant of the *Q*-factor obtained as a function of asymmetry parameter is largest at the NIR Fano resonance frequency and then decreases (with saturation) towards THz Fano resonance frequency. Both distributions display similar trend which tend towards an exponential decay behaviour.

## Methods

**Fig. 1:**
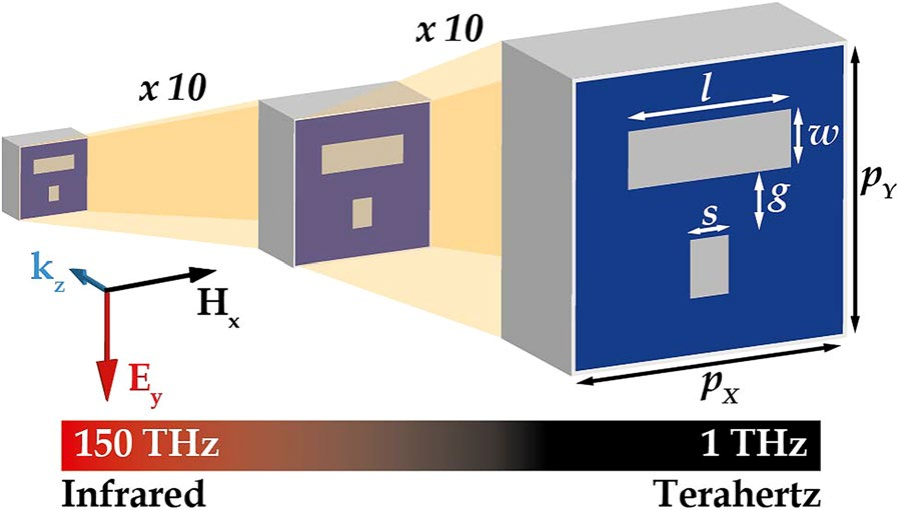
Schematic illustration of the asymmetric dipole bars with the dimensions enlarged proportionally from NIR to THz regime. The electric field and magnetic field are polarized along the *y*- and *x*-axis respectively, and *k*_*z*_ is the propagation direction of the incident plane wave source

For the choice of simplicity, but not limited to this design of metamaterial structure, we evaluated the *Q*-factors of complementary asymmetric dipole bars to elucidate the behaviour of *Q*-factors from NIR to THz regime. In Fig. 1, the dimensions of a unit cell of the asymmetric dipole bars in THz regime are illustrated as follow: both periods, *p*_*x*_ and *p*_*y*_ of the unit cell is 70 µm; the width *w* and gap between the dipole bars *g* are each 14 µm; the long bar length *l* is fixed at 42 µm and the short bar length *s* varies from 10 to 42 µm which evolves the unit cell from asymmetric to symmetric structure. The thickness of the aluminium metal is kept constant at 40 nm. Subsequently, taking the dimensions of the asymmetric dipole bars in the THz regime as a reference, the dimensions of the asymmetric dipole bars from NIR to mid-IR regimes are reduced proportionally by 100 and 10 times, respectively. In all cases, the asymmetry parameter of the Fano resonators is defined as the ratio between the difference in the length of long and short dipole bars to the length of the long dipole bar, $\alpha = \frac{l - s}{l}$.

Numerical simulations based on finite-differential time-domain (FDTD) technique were performed using commercially available electromagnetic simulation software, computer simulation technique (CST) Microwave studio using the frequency domain solver. Unit cell boundary conditions were imposed in the periodic directions of *x* and *y*, and the incident plane wave source is excited from the *Z*_max_ direction with the electric field polarized along the symmetry axis of the asymmetric dipole bars as shown in Fig. 1. Throughout the interested regions of the electromagnetic spectrum, the quartz substrate is modelled as loss free and assigned with a permittivity of 2.25. The aluminium is modelled as a lossy metal in the THz regime with DC conductivity of *σ* = 3.56 × ×10^7^ S/m, and permittivity values of aluminium in the optical regime were taken from the data measured by Palik [36].

## Results and discussion

**Fig. 2:**
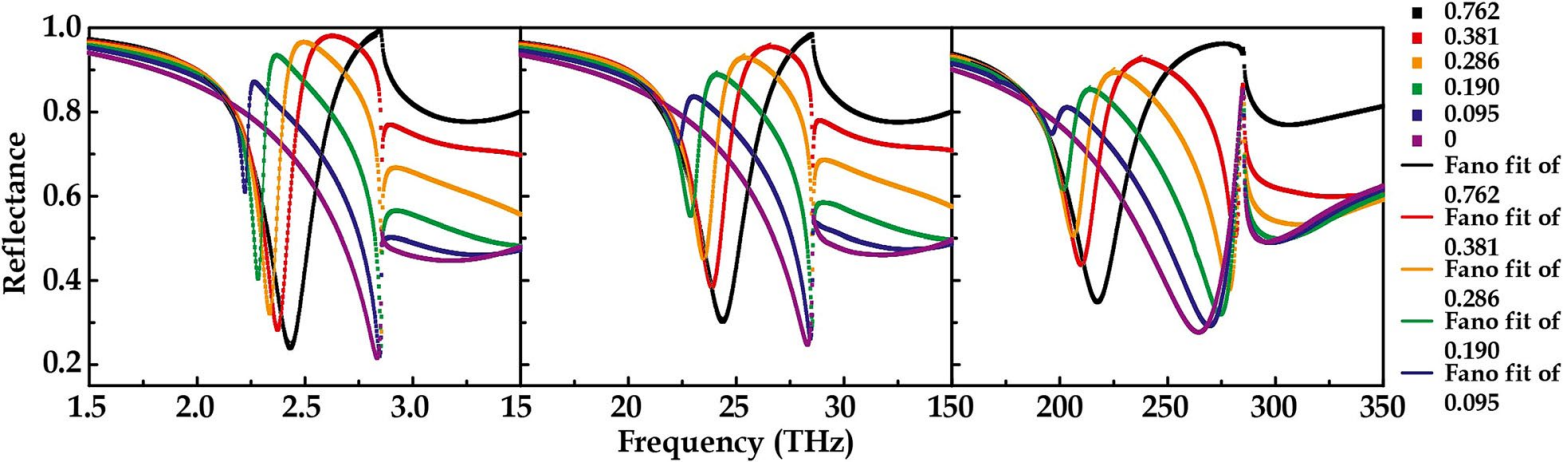
Simulated reflectance spectra of the asymmetric dipole bars at different regimes of the electromagnetic spectrum - NIR to THz (right to left). Coloured solid lines represent the Fano lineshape fitted to Eq. 1 at different asymmetry parameters

In Fig. 2, the simulated reflectance spectra of the asymmetric dipole bars show that the symmetric dipole bars produce a broad dipole resonance accompanied by a lattice resonance at the higher frequency of each frequency regime. The broad dipole resonance at 264 THz is more pronounced in the NIR regime. For asymmetric dipole bars, the length of the short bar, *s* is reduced relative to the length of the long bar, *l*. When the symmetry of the dipole bars is broken, two resonances are observed from the spectrum: the Fano resonance is distinct as a sharp asymmetric lineshape at a lower frequency, associated with the background of the broad dipole resonance. A further decrease in the length of the short bar will increase the asymmetry parameters from 0 to 0.762, and a noticeable blue-shift of the Fano resonance is observed.

**Fig. 3:**
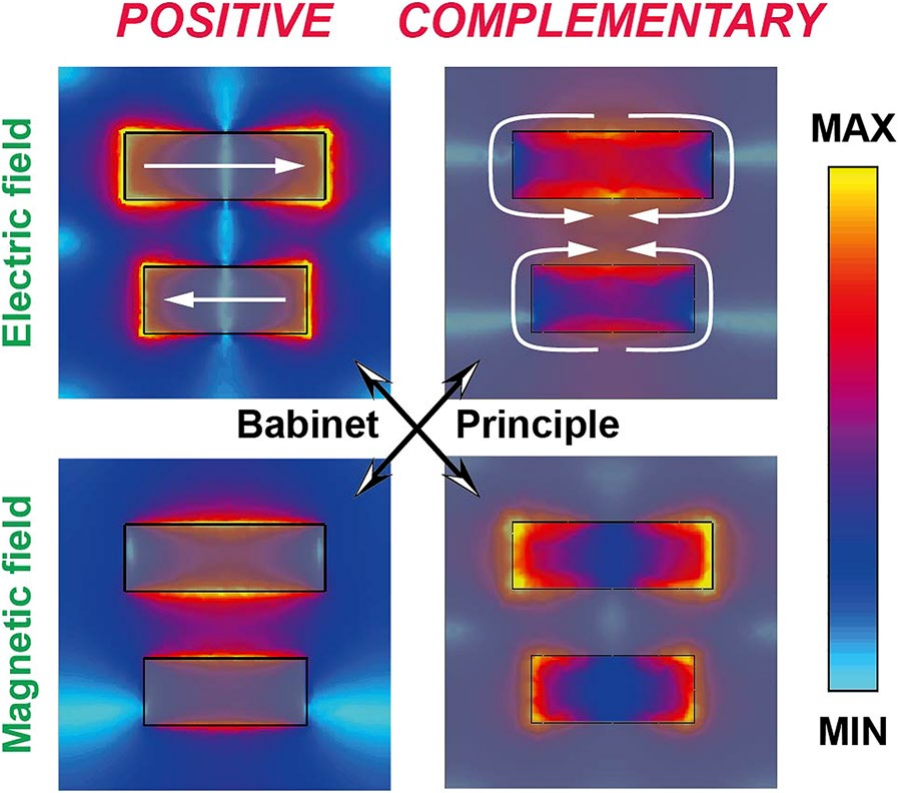
Simulated electric and magnetic field distributions of the positive and complementary asymmetric dipole bars at asymmetry parameter of 0.190 which validate the Babinet’s Principle for electromagnetic fields. The maximum electric and magnetic field strengths of the positive and complementary asymmetric dipole bars are (2.6 × 10^7^ V/m and 5 × 10^4^ A/m) and (1 × 10^7^ V/m and 5.5 × 10^4^ A/m), respectively. The white solid arrows indicate the direction of induced surface currents on the aluminium metal

Unlike Fano resonance in positive structures, which appears as a sharp dip in the transmission spectrum, the Fano resonance of complementary asymmetric dipole bars appear as a sharp dip in the reflectance spectrum. Simulated electric and magnetic field distributions of the positive and complementary asymmetric dipole bars are shown in Fig. 3 to better comprehend the spectral feature of the Fano resonance. In positive asymmetric dipole bars, strong enhancement of the electric field occurs at both ends of the dipole bars whereby the charges in each dipole bar flow opposite to one another, inducing anti-parallel surface currents which is representative of an electric quadrupole mode. In complementary asymmetric dipole bars, out-of-phase circular surface currents are induced outside each bar which excite the magnetic quadrupole mode, and electric fields are confined at the inner top and bottom sides of each dipole bar, analogous to the magnetic field distribution in the positive asymmetric dipole bars. The destructive interference of the quadrupole mode (dark mode) with the dipole mode (bright mode) contributes to the origin of the Fano resonance. Similarly, the electric field distribution of the positive asymmetric dipole bars relates very well with the magnetic field distribution of the complementary asymmetric dipole bars. This behaviour demonstrates strong coherence with the Babinet’s Principle for electromagnetic fields [37]. In both positive and complementary asymmetric dipole bars, the direction of the flow of induced surface currents and position of the field distribution match very closely to the polarization of the electric field and magnetic field incident onto the metamaterial structures.

**Fig. 4:**
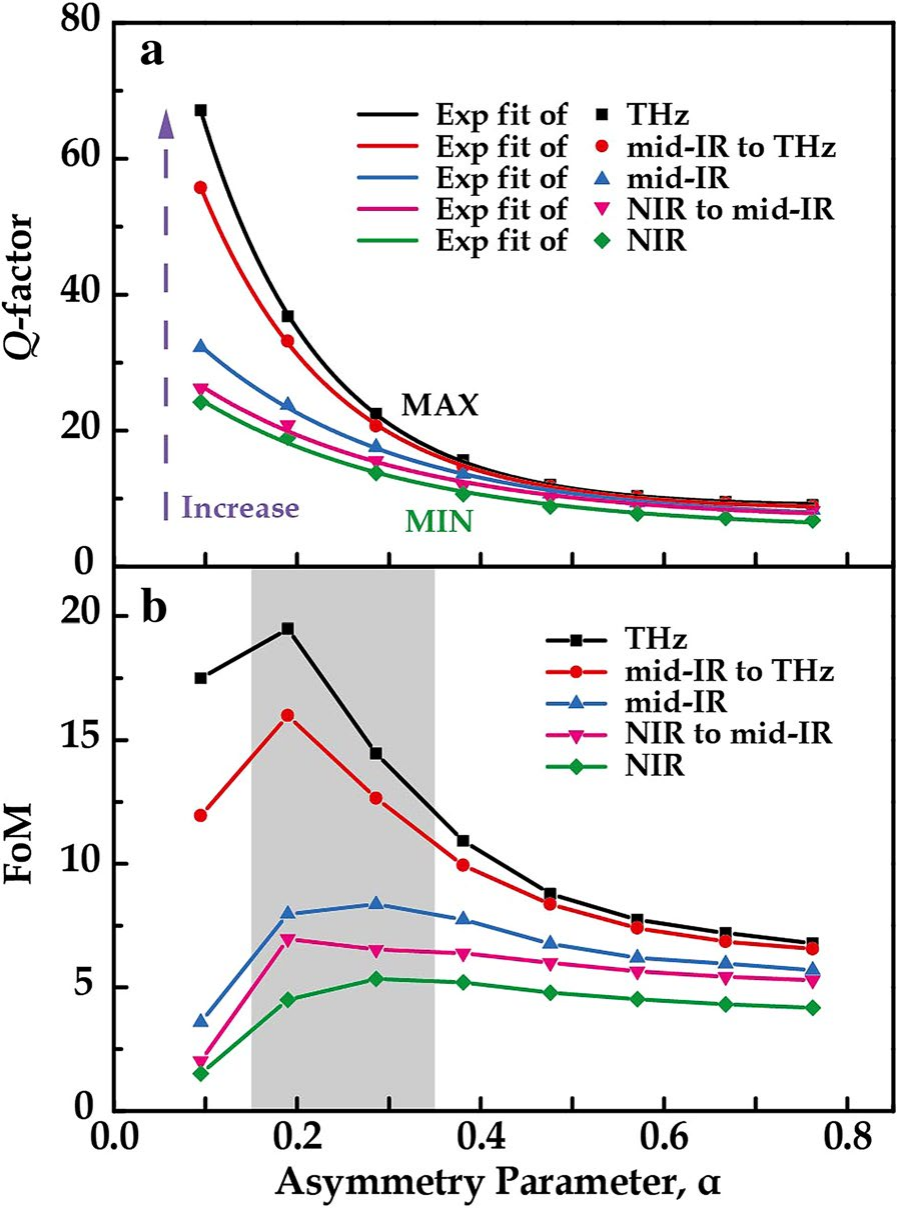
**a** Numerically evaluated *Q*-factor and, **b** FoM plotted against the asymmetry parameter. The decay of the *Q*-factors with increasing asymmetry parameter at different regimes was fitted with an exponential decay function

Since the dark mode is subradiant, radiative losses in Fano resonance are effectively suppressed which results in a narrow linewidth and high *Q*-factor. The *Q*-factors of the simulated reflectance spectra in the NIR to THz regime were evaluated from parameters obtained through fitting with the Fano resonance lineshape equation [38]:

1 $$R = A_{0} + F_{0} \frac{{\left[ {q + \frac{{2\left( {\omega - \omega_{0} } \right)}}{\varGamma }} \right]^{2} }}{{1 + \left[ {\frac{{2\left( {\omega - \omega_{0} } \right)}}{\varGamma }} \right]^{2} }}$$

where *A*_0_ and *F*_0_ are constant factors, *q* is the asymmetry parameter (different from the asymmetry parameter, *α* of the Fano structures) which determines the ratio between resonant and non-resonant state, *ω*_0_ is the resonance frequency and *Γ* is the full-width at half maximum of the resonance frequency. In the context of this work, *Q*-factor is defined as the ratio between the resonance frequency and the full-width at half maximum of the resonance dip in the reflectance spectrum. As shown in Fig. 4a, the *Q*-factor at different regimes is plotted as a function of asymmetry parameter, *α*. The increase in the *Q*-factor from NIR to THz regime reveals that the radiative losses at the lower frequency as compared to higher frequency are more effectively suppressed. In addition, the size of the Fano resonators at NIR frequencies is less than half the resonance wavelength, which suggests that majority of the energy is stored as kinetic energy, and lost through electron scattering in metals at femtosecond scale [18]. Despite the higher *Q*-factor at lower asymmetry parameter, the performance of the Fano resonators is limited by the intensity of the Fano resonance dip. There exists a trade-off between the strength of the Fano resonance and the *Q*-factor. Therefore, a Figure of Merit (FoM) is proposed to quantify the amount of losses which can be reduced such that the strength of the Fano resonance is still well-defined with maximum field confinement [35]. The FoM is defined as the product of the *Q*-factor and the resonant intensity, with the resonant intensity being the dip-to-peak amplitude of the Fano resonance frequency. As shown in Fig. 4b, the grey shaded area indicates the sector in which the performance of the Fano resonator is optimized for narrow linewidth and strong resonant lineshape across the NIR to THz regime.

**Fig. 5:**
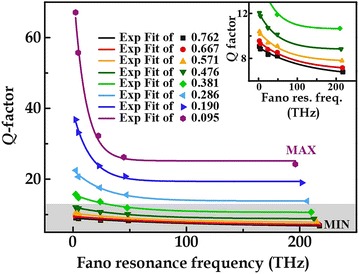
Numerically evaluated *Q*-factor plotted against the Fano resonance frequency which is fitted with an exponential decay function. The inset shows the magnified view of the grey shaded area where the plots for higher asymmetry parameters are presented

In conjunction with Fig. 4a, the *Q*-factor as a function of Fano resonance frequency across the NIR to THz regime was also plotted as shown in Fig. 5 to fully visualize the decay trend of *Q*-factors under different situations. As expected, the *Q*-factor is larger at THz Fano resonance frequency, with it being highest at the lowest asymmetry parameter. It is evident from both Figs. 4a, 5 that the decreasing trend of the *Q*-factor exhibits an exponential decay feature. Therefore, to visualize the decay trend of *Q*-factor against asymmetry parameter or Fano resonance frequency, a systematic study of the decay constants is investigated.

**Fig. 6:**
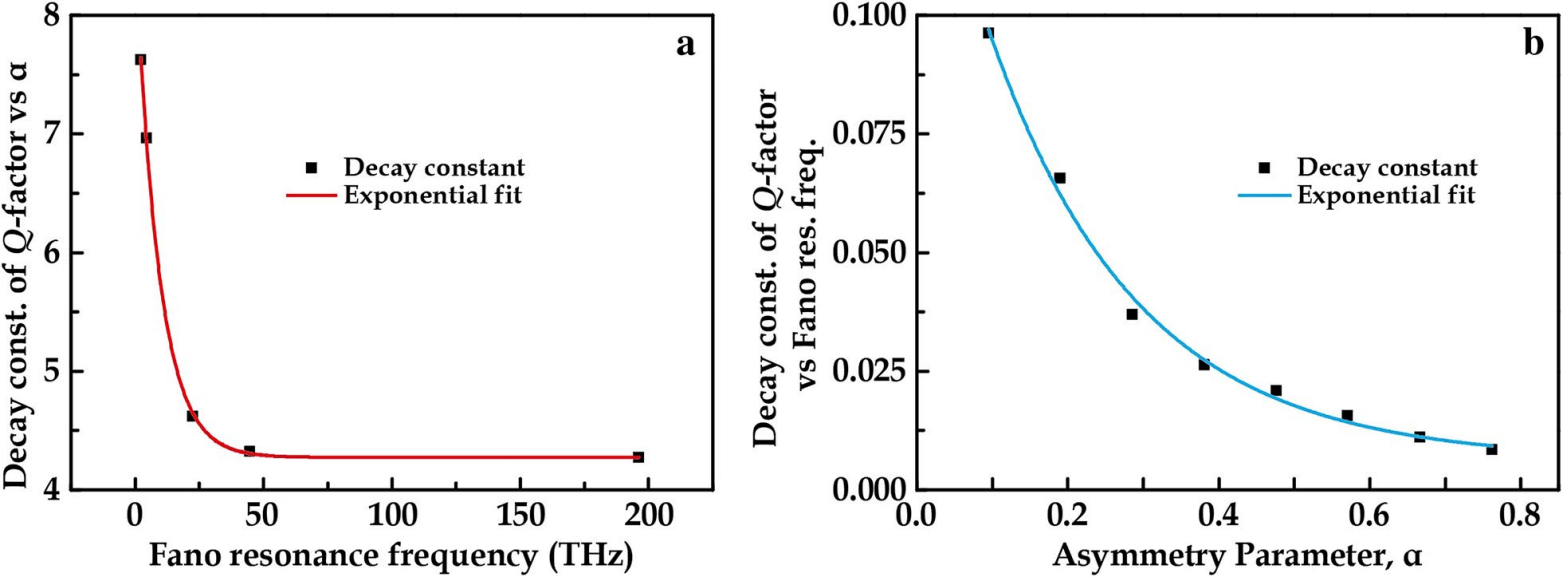
Decay constants (const.) evaluated from *Q*-factor against—**a** asymmetry parameter which is plotted against Fano resonance frequency, and **b** Fano resonance frequency which is plotted against asymmetry parameter

Technically, *Q*-factor means the ratio between the amount of energy stored to the amount of energy lost through dissipation in the resonator system. Therefore, the decay constants that are obtained through exponential fitting under different asymmetry parameter and Fano resonance frequency describe the rate at which the energy retained is dependent on the geometrical and frequency factors, respectively. An exponential decay function was fitted to various sets of *Q*-factor, and the decay constants obtained further demonstrate an exponential decay behaviour as depicted in Fig. 6. Both Fig. 6a, b constitute as a set of standard system for tailoring and optimizing the dimensions of the Fano resonators which are complemented by Figs. 4, 5 as calibration plots. The calibration plots need to have information at the extremities of the interested frequency or asymmetry parameter so that any new data points can be interpolated from within the range of available data points. As an example, to find out the *Q*-factor of a Fano resonator near 150 THz with any asymmetry parameters, decay constant from Fig. 6a is extracted and used to plot the *Q*-factor in any positions of Fig. 4a. Next, since the *Q*-factors are unknown, maximum and minimum *Q*-factors at 150 THz are obtained from the calibration plot of Fig. 5 to allow full prediction on the behaviour of the *Q*-factor of Fano resonators near 150 THz with varying asymmetry parameter. This works similarly for estimating the *Q*-factor of Fano resonators at a specific asymmetry parameter across the NIR to THz regime.

## Conclusions

In conclusion, the universal behaviour of Fano resonance in symmetry-broken resonators is investigated in asymmetric dipole bars. Sharp asymmetric and strong resonant lineshape were observed with *Q*-factor of at least 20 from the near-infrared to terahertz regime. Positive and complementary structures follow closely to the Babinet’s Principle for electromagnetic fields. A standard system based on exponential decay function has been developed to characterize the behaviour of *Q*-factor under varying asymmetry parameter or Fano resonance frequency. Decay constants obtained from the exponential fittings provide insights into the rate at which energy is stored under different conditions. This work will be more extensive if a larger superset can be established based on varying materials or geometries, and further evaluated based on their decay trends. The process may be tedious but once a standard system is developed, it would enable smart designing of the metamaterial structure to tailor its *Q*-factor by itself through machine learning techniques. The standard system is beneficial and useful for the realization of scalable and “smart” metadevices across a wide spectral range.
